# Tangshen Formula Attenuates Colonic Structure Remodeling in Type 2 Diabetic Rats

**DOI:** 10.1155/2017/4064156

**Published:** 2017-02-20

**Authors:** Pengmin Chen, Jingbo Zhao, Haojun Zhang, Xin Yang, Tingting Zhao, Huicun Zhang, Meihua Yan, Lin Pan, Xin Li, Yun Zhang, Ping Li

**Affiliations:** ^1^Beijing Key Lab for Immune-Mediated Inflammatory Diseases, Institute of Clinical Medical Science, China-Japan Friendship Hospital, Beijing, China; ^2^Department of Clinical Medicine, Aarhus University, Aarhus, Denmark; ^3^Department of Combination of Traditional Chinese and Western Medicine, Beijing Hospital of Traditional Chinese Medicine, Capital Medical University, Beijing 100010, China

## Abstract

*Aim*. This study investigated the effect and mechanism of the Chinese herbal medicine Tangshen Formula (TSF) on GI structure remodeling in the rat model of diabetes.* Methods*. Type 2 diabetic rats were used. Wet weight per unit length, layer thicknesses, levels of collagens I and III, nuclear factor kappa B (NF-*κ*B), interferon-*γ* (IFN-*γ*), interleukin-6 (IL-6), transforming growth factor-*β*1 (TGF-*β*1), and Smad2/3 expression in the rat colon were measured.* Results*. Compared with the control group animals, wet weight and layer thicknesses of the colon increased, and expressions of collagens I and III, NF-*κ*B, IFN-*γ*, IL-6, TGF-*β*1, and Smad2/3 increased significantly in the diabetic animals. TSF inhibited increase in colonic wet weight and layer thicknesses, downregulated expressions of collagens I and III in the mucosal layer, and downregulated expressions of NF-*κ*B, IFN-*γ*, IL-6, TGF-*β*1, and Smad2/3 in the colon wall. Furthermore, level of expression of NF-*κ*B was associated with those of TGF-*β*1 and Smad2/3. Expression of TGF-*β*1 was associated with the most histomorphometric parameters including colonic weight, mucosal and muscle thicknesses, and levels of collagens I and III in mucosal layer.* Conclusion*. TSF appears to attenuate colonic structure remodeling in type 2 diabetic rats through inhibiting the overactivated pathway of NF-*κ*B, thus reducing expressions of TGF-*β*1.

## 1. Introduction

People with diabetes mellitus (DM) often have gastrointestinal (GI) motility disorders. Diabetes can affect the entire GI tract from the esophagus to the anorectal region. Symptoms may include dysphagia, early satiety, reflux, nausea and vomiting, constipation, or diarrhea [1–3]. Disorders of transit and motility in the colon [4–6] likely contribute to constipation in diabetes [7]. It is well known that in diabetes the muscle and mucosal layers, functions of neurons and interstitial cells of Cajal, and GI hormone levels are impaired [1, 8]. Although dysfunctions of neurons and interstitial cells of Cajal in diabetes seem to be mainly responsible for GI motility disorder, the role of muscle and mucosal layer remodeling in GI motility remains unclear and needs to be investigated further. How diabetic enteropathy affects the colon is also not entirely understood [1]. Histomorphologic remodeling of the colon wall may be involved in the pathogenesis of colon motility dysfunction [9].

Our previous studies demonstrated that, in diabetic rats of either type 1 or type 2, total wall thickness, muscle layer thickness, and weight per unit length of the colon significantly increased compared with nondiabetic rats [10–12]. Furthermore, biomechanical remodeling of the colon wall also occurred concomitant to increase in wall stiffness in the diabetic colon [10–12]. The molecular mechanism of colon thickening and stiffening in diabetes remains unclear. It is possible that since diabetes, especially type 2 diabetes, is a chronic inflammatory disease, proinflammatory factors are involved [13]. To the best of our knowledge, there is no medicine that addresses diabetic enteropathy by targeting tissue structural remodeling including proliferation of the GI wall.

Tangshen Formula (TSF) is a traditional Chinese herbal compound. Our previous study found that TSF could reduce glomerulosclerotic index and renal tubular interstitial fibrosis in type 1 and type 2 diabetic rats through inhibiting overexpression of transforming growth factor-*β*1 (TGF-*β*1) [14]. Another investigation of ours indicated that TSF could inhibit expressions of nuclear factor kappa B (NF-*κ*B) and proinflammatory cytokines, such as tumor necrosis factor- (TNF-) *α*, interleukin-1 (IL-1), and monocyte chemoattractant protein-1 (MCP-1), in the kidney of diabetic rats [15]. Therefore, the aim of this study is to explore whether TSF attenuates changes of diabetes-induced GI wall structure through inhibiting proinflammatory factors in type 2 diabetic rats.

## 2. Materials and Methods

### 2.1. Reagents

Rabbit polyclonal antibodies against TGF-*β*1 (cat. 3711), Smad2/3 (cat. 5678), and NF-*κ*B (cat. 8242) were purchased from Cell Signaling Technology (Danvers, MA, USA). Other reagents were rabbit polyclonal antibodies against collagen I (cat. ab34710), collagen III (cat. ab7778), and *β*-actin (cat. ab129348) from Abcam (Cambridge, MA, USA) and against interferon-*γ* (IFN-*γ*; cat. PB0062) and interleukin-6 (IL-6; cat. PB0061) from Boster Biological Technology (Wuhan, China); Polink-2 Plus HRP Rabbit with DAB Kit (cat. D39-6) from GBI Labs (Bothell, WA, USA); Peroxidase Affinipure Goat Anti-Rabbit IgG (cat. 111-035-144) from Jackson ImmunoResearch (West Grove, PA, USA); Mayer's hematoxylin (cat. S3309) from Dako (Glostrup, Denmark); bovine serum albumin (BSA; cat. C2312) and defat dried milk (cat. P1622) from Applygen Technologies (Beijing, China); diaminobenzidine (DAB) kit (cat. AR1022) from Boster Biological Technology (Wuhan, China); streptozotocin (STZ; cat. 0130) from Sigma-Aldrich (Shanghai, China); and ethanol (cat. 10009218), methanol (cat. 10014118), and xylene (cat. 10023418) from Sinopharm Chemical Reagent Co. Ltd. (Shanghai, China). Standard diet and high-fat diet were purchased from Beijing HFK Bioscience (Beijing, China).

### 2.2. Composition and Preparation of Tangshen Formula

TSF is composed of 7 natural herbs: 35.3% astragalus root (*Astragalus membranaceus* (Fisch.) Bge.), 17.6% burning bush twig (*Euonymus alatus* (Thunb.) Sieb.), 14.1% rehmannia root (*Rehmannia glutinosa *Libosch.), 11.8% bitter orange (*Citrus aurantium *L.), 10.6% cornus fruit (*Cornus officinalis* Sieb. et Zucc.), 7.1% rhubarb root and rhizome (*Rheum palmatum* L.), and 3.5% notoginseng root (*Panax notoginseng* (Burk.) F. H. Chen). TSF was prepared by Jiangyin Tianjiang Pharmaceutical (Jiangsu, China). Briefly, the manufacturing process involved soaking (30 minutes) and boiling (60 minutes) the herbs in distilled water, extracting with water twice, filtering and condensing the decoction to 1 g/mL, and then spray drying into fine granules [16]. Quality control was accomplished by analyzing the resultant chemical composition of TSF using high-performance liquid chromatography/mass spectrometry (HPLC/MS).

### 2.3. Animal Model

This study was approved by the Ethics Review Committee for Animal Experimentation of the Institute of Clinical Medicine, China-Japan Friendship Hospital, Beijing, and was performed in accordance with the* Guiding Principles for the Care and Use of Laboratory Animals* (2012-A04).

Fifty male 8-week-old Wistar rats, weighing about 200 g, were purchased from Beijing Laboratory Animal Research Center (Beijing, China). After adaptive feeding for 3 days, the animals were randomly divided into 2 groups. The normal control group (NC, *n* = 10) were fed a standard diet. The high-fat diet group (*n* = 40) were fed a diet consisting of 38% fat, 12% protein, and 50% carbohydrate until termination of experiment. All rats were housed under a 12-hour light-dark cycle and allowed free access to food and water. At the end of 6 weeks of feeding, the insulin clamp test was used to check glucose infusion rate in 4 rats each from the NC and high-fat diet groups. Infusion rate in the high-fat diet group was found to be much lower than in the NC group, indicating that insulin resistance was successfully established in the high-fat diet animals. To induce diabetes, these rats were then injected once intraperitoneally with 30 mg/kg streptozotocin (STZ), which was dissolved in citrate buffer (0.01 M, pH 4.3). Rats in the NC group were each injected once intraperitoneally with citrate buffer at equal volume. Fasting blood glucose was measured in the high-fat diet group 3 days after STZ injection. Rats with blood glucose higher than 11.7 mmol/L were divided into 2 groups: diabetes mellitus group (DM, *n* = 15) and Tangshen Formula group (TSF, *n* = 15). Animals in the TSF group were intragastrically administered Tangshen Formula at a dose of 2.4 g/kg, which was dissolved in saline. Animals in the NC and DM groups were intragastrically administered saline. Tangshen Formula and saline were given once daily over 20 weeks. Body weight and fasting blood glucose were measured every 2 and 4 weeks, respectively, until termination of experiment.

### 2.4. Sample Collection

At the end of the 27-week experiment, rats in all groups (10 for each group) were anesthetized by intraperitoneal injection of chloral hydrate and euthanized by exsanguination. A 5 cm segment of colon was excised 5 cm distal to the ileocecal valve. Residual contents in the lumen were gently cleared using prechilled saline. After weighing the segment, 3 cm of distal segment was snap-frozen in liquid nitrogen. The remaining 2 cm segment was fixed in 10% phosphate-buffered formalin for 24 hours.

### 2.5. General Histologic Staining

Next, the samples were embedded in paraffin and 5 *μ*m sections were then cut perpendicular to the mucosal surface. The sections were stained with hematoxylin and eosin (HE). Layer thickness was measured using Sigmascan Pro 4.0 software (Informer Technologies, Madrid, Spain) and the number of smooth cells in circular and longitudinal layers per unit area was manually counted by counting nucleus number using Image-Pro Plus 6.0 software (Media Cybernetics, Rockville, MD, USA).

### 2.6. Immunohistochemical Staining for Collagens I and III

Staining for collagens I and III was then performed. Endogenous peroxidase was quenched with 3% H_2_O_2_ in ethanol for 15 minutes at room temperature. Antigen retrieval was performed by boiling sections in 10 mM citrate buffer (pH 6.0) for 18 minutes. Nonspecific staining was blocked by incubating sections in 5% BSA-PBS buffer (room temperature, 30 minutes). The sections were then incubated with primary antibody (rabbit IgG against collagen I or collagen III of rats; 1 : 200, diluted using 1% BSA-PBS) or normal rabbit IgG (200 *μ*g/mL) pretreated with excessive collagen I or collagen III (1 : 200, diluted in 1% BSA-PBS, negative control) at 4°C overnight. Complete washing was performed using 0.05% tween-20-PBS, after which sections were incubated with biotinylated secondary antibodies and then with streptavidin-peroxidase secondary antibodies at room temperature for 20 minutes. The positive immunostaining in the tissues was visualized by using 3,3′-diaminobenzidine tetrahydrochloride (DAB) and counter staining with Mayer's hematoxylin.

### 2.7. Image Analysis

For analysis of collagens I and III, 6 photomicrographs of the muscle and mucosa locations of the same layer in each slide were taken randomly. Unnecessary area of photomicrographs was cut out using paint software. The area of yellow-brown was defined as immune-positive areas and measured by Image-Pro Plus 6.0 software (Media Cybernetics, Rockville, MD, USA). The fractions of collagens I and III in mucosa and muscle layers were computed as follows: fraction of collagen I or collagen III = immunopositive area/total area of measurement. For counting the number of smooth muscle cells in circular and longitudinal layers per unit area, 6 photomicrographs of the muscle layer in each slide were taken randomly. The area of smooth muscle layer was measured using Image-Pro Plus 6.0 software and the number of smooth muscle cells was manually counted.

### 2.8. Western Blot

Levels of TGF-*β*1, Smad2/3, NF-*κ*B, IFN-*γ*, and IL-6 expression in full thickness were analyzed using Western blot.

#### 2.8.1. Protein Extraction

The tissue was homogenized in RIPA lysis buffer (50 mM Tris-HCl, pH 7.5, 150 mM NaCl, 1% NP-40, 0.25% sodium deoxycholate, 1 mM EDTA Na_4_, 1 mM beta-glycerophosphate, 2.5 mM sodium pyrophosphate, 1 mM Na_3_VO_4_, 10 mM NaF, 1 mM PMSF, and 1x cocktail). The homogenate was centrifuged at 12000*g* for 10 minutes at 4°C and the supernatant was collected. Protein concentration was determined by the Lowry protein assay.

#### 2.8.2. Western Blot Analysis

The protein samples were denatured by boiling for 5 minutes in SDS sample buffer. Afterward, 40 *μ*g of protein per lane was electrophoretically resolved with 12% SDS-PAGE and transferred to polyvinylidene difluoride (PVDF) membranes by electroblotting in transfer buffer (25 mM Tris-base, 192 mM glycine, and 20% methanol). The membranes were incubated with 5% nonfat milk at room temperature for 1 hour and then incubated with primary antibody (rabbit polyclonal antibody against TGF-*β*1, Smad2/3, NF-*κ*B, IFN-*γ*, IL-6, or *β*-actin dissolved with 1% nonfat milk) with gentle agitation at 4°C overnight. After washing with 0.05% tween-20-TBS thrice, 10 minutes per washing, the membrane was incubated with secondary antibody at room temperature for 90 minutes. The membrane was then washed 4 times using the same buffer, after which immunostaining was visualized using enhanced chemiluminescence reagent according to the manufacturer's instructions. Intensities of the protein bands were scanned using Image j 1.44 software (National Institutes of Health, Bethesda, MD, USA).

### 2.9. Statistical Analysis

Results were expressed as mean (SD) unless indicated in the text. Differences were tested using* t*-test and ANOVA. In linear regression analysis, Spearman's Rank Correlation was used to demonstrate the possible association between NF-*κ*B and inflammatory cytokines as well as TGF-*β*1 and morphologic changes of the colon. Results were regarded as significant when *P* < 0.05.

## 3. Results

### 3.1. Effect of TSF on Body Weight and Fasting Blood Glucose

**Figure 1:**
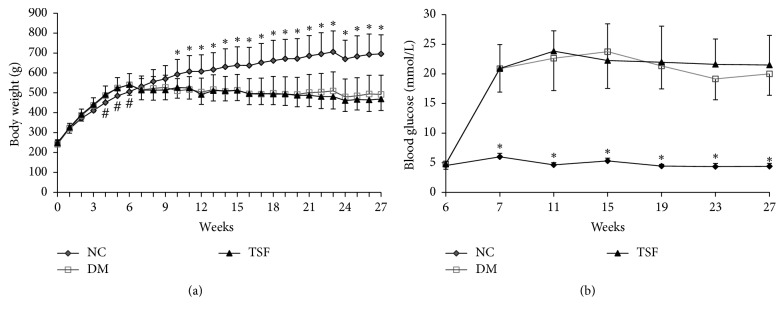
Effect of TSF on body weight (a) and blood glucose (b). Tangshen Formula was administered to the TSF group rats from week 7, that is, 3 days after STZ administration through week 27. Values expressed as mean (SD), *n* = 9 (^#^*P* < 0.05, ^*∗*^*P* < 0.01 compared with DM or TSF group). DM: diabetic group; NC: normal control group; TSF: Tangshen Formula group.

After 6 weeks on their respective diets, body weight (Figure 1(a)) increased more rapidly in the high-fat diet group (DM and TSF) than in the NC group, with significant difference at 4 through 6 weeks (*P* < 0.05). After STZ or citrate buffer administration at week 6, body weight in DM and TSF groups decreased (*P* < 0.05) and then maintained until the end of the experiment, with no significant difference between the 2 groups. However, body weight in the NC rats increased gradually through week 23 and then maintained until the end of the experiment. Marked differences in body weight between the NC and DM groups and between the NC and TSF groups were evident at week 9, which was 3 weeks after administering citrate buffer or STZ (*P* < 0.01) and continued through the end of the experiment.

Fasting blood glucose levels (Figure 1(b)) did not differ between the NC and high-fat diet groups before and after 6 weeks on their respective diets. After STZ or citrate buffer administration, fasting blood glucose in the TSF and DM groups significantly increased and reached to peak after 3 days and then maintained until the end of experiment. Fasting blood glucose in the NC group did not change markedly throughout the experiment.

### 3.2. Effect of TSF on Morphologic Structure of Colon

#### 3.2.1. Wet Weight and Wall Thickness of Colon

**Figure 2:**
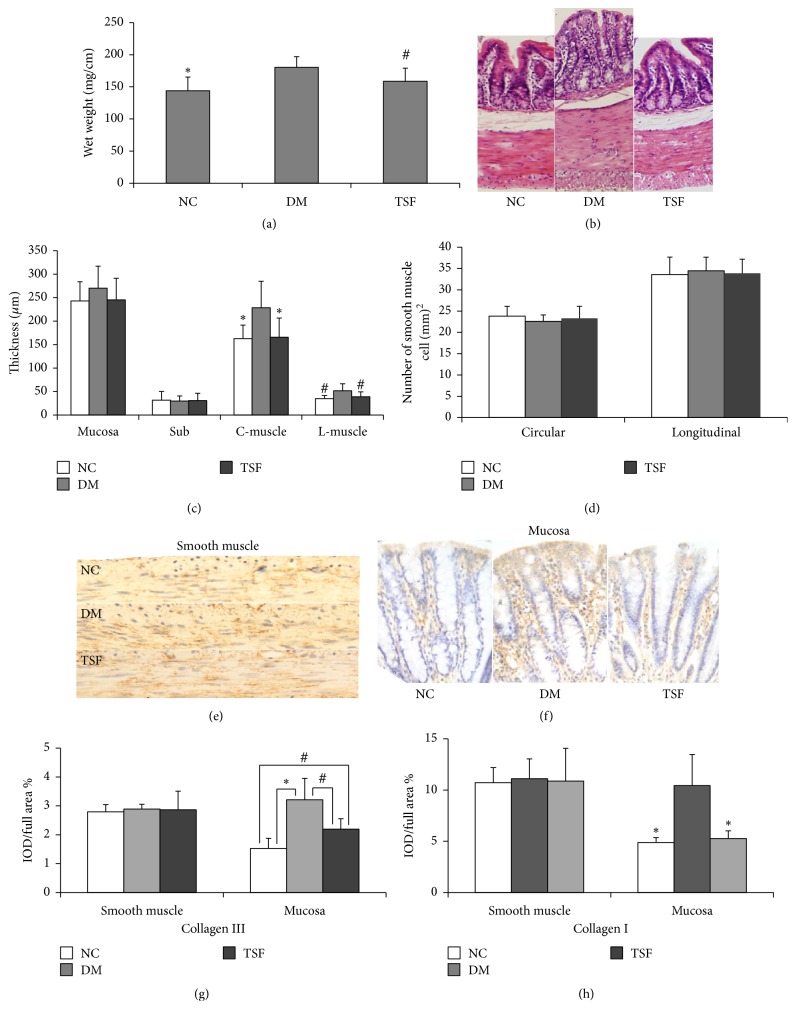
Effect of TSF on the morphologic structure of colon. (a) Wet weight per unit length; (b) HE staining; (c) comparison of wall thickness among groups; (d) comparison of smooth muscle cells number per unit area among groups; (e) collagen III immunostaining in smooth muscle; (f) collagen III immunostaining in mucosa layer; (g) comparison of collagen III contents among groups; (h) comparisons of collagen I contents among groups. Values are expressed as mean (SD), *n* = 6 for each group (^*∗*^*P* < 0.01, ^#^*P* < 0.05 compared with DM group). C-muscle: circular muscle layer; DM: diabetic group; IOD: integral optical density; L-muscle: longitudinal muscle layer; NC: normal control group; Sub: submucosa; TSF: Tangshen Formula group.

Compared with the NC group, colonic wet weight per unit length increased in the DM and TSF groups. However, the extent of increase was lower in the TSF group than in the DM group. Significant difference was found between the DM and TSF groups (*P* < 0.05), but not between the TSF and NC groups (*P* > 0.05) (Figure 2(a)).

Compared with the NC group, muscle thickness increased in the longitudinal and circular smooth muscle layers as well as the mucosal layer in the DM group. In the TSF group, however, increase in layer thickness was inhibited. Significant differences were found between the DM and TSF groups in thicknesses of longitudinal and circular smooth muscle layers (*P* < 0.05). No marked differences were found for other layers (*P* > 0.05) (Figures 2(b) and 2(c)).

#### 3.2.2. The Number of Smooth Muscle Cells in Smooth Muscle Layer per Unit Area in the Colon

As shown in Figure 2(d), the number of smooth muscle cells per unit area in both circular and longitudinal smooth muscle layers did not significantly change among three groups.

#### 3.2.3. Effect of TSF on Distributions of Collagen I and Collagen III in the Colon

Immune-positive areas of collagens I and III in the colon samples were yellow-brown (Figures 2(e) and 2(f)). Such color was not evident in the negative control, demonstrating that the stain was specific for collagens I and III. In the colon, distributions of collagens I and III were similar in that they were found in all layers.

In the muscle layer, distributions of both collagen types were dispersed and inhomogeneous (Figure 2(e)). Compared with the NC group, intensities of immunostaining for collagen I (Figure 2(h)) and collagen III (Figure 2(g)) were unchanged in the DM and TSF groups.

In the mucosal layer, distributions of collagens I and III were mainly limited to the lamina propria (Figure 2(f)). Compared with the NC group, immunostaining intensities markedly increased in the DM group for both collagens I and III. In the TSF group, there was an obvious increase for collagen III; however the extent of the increase was less than that of DM group (Figure 2(g)), and there was almost no increase for collagen I (Figure 2(h)). Significant differences in distributions of collagens I and III were found between the NC and DM groups as well as between the DM and TSF groups (*P* < 0.01 and *P* < 0.05, resp.). Significant difference in distribution of collagen III was also found between NC and TSF groups (*P* < 0.05). No marked difference was found between the NC and TSF groups for distribution of collagen I (*P* > 0.05).

### 3.3. Effect of TSF on Levels of NF-*κ*B, IFN-*γ*, and IL-6 Expression in Colon

**Figure 3:**
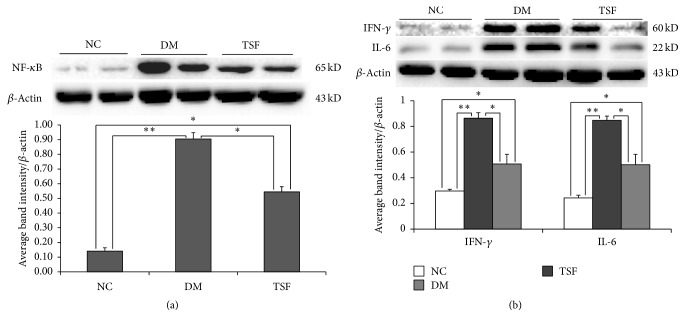
Western blotting results for (a) NF-*κ*B, (b) IFN-*γ*, and IL-6 expression in colon samples. Values are expressed as mean (SD), *n* = 6 for each group (^*∗*^*P* < 0.01, ^*∗∗*^*P* < 0.001). DM: diabetic group; NC: normal control group; TSF: Tangshen Formula group.

Band only appeared at the location of target protein, demonstrating that the stain was specific. Levels of NF-*κ*B, IFN-*γ*, and IL-6 expression markedly increased in the DM group. However, increased expression was inhibited in the TSF group (Figure 3). Significant differences in expressions of these proteins were found between the NC and DM, DM and TSF, and NC and TSF groups (all *P* < 0.01).

### 3.4. Effect of TSF on Levels of TGF-*β*1 and Smad2/3 Expression in Colon

**Figure 4:**
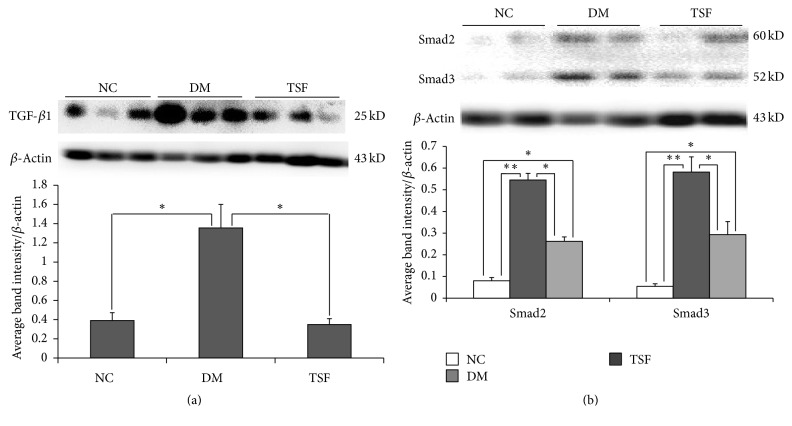
Western blotting results for (a) TGF-*β*1; (b) Smad2 and Smad3 expression in colon samples. Values are expressed as mean (SD), *n* = 6 for each group (^*∗*^*P* < 0.01, ^*∗∗*^*P* < 0.001). DM: Diabetic group; NC: normal control group; TSF: Tangshen Formula group.

Band only appeared at the location of target protein, demonstrating that the stain was specific. Level of TGF-*β*1 expression markedly increased in the DM group but inhibited the TSF group (Figure 4(a)). Significant difference in levels of TGF-*β*1 expression was found between the NC and DM groups (*P* < 0.01) as well as between the DM and TSF groups (*P* < 0.01). No significant difference was found between the NC and TSF groups (*P* > 0.05).

Compared with the NC group, expressions of Smad2 and Smad3 increased significantly in the DM and TSF groups (Figure 4(b)). However, the extent of increase was much less in the TSF group than DM group. Marked differences in expressions of Smad2 and Smad3 were found between the NC and DM groups (*P* < 0.001), the NC and TSF groups (*P* < 0.01), and the DM and TSF groups (*P* < 0.01).

### 3.5. Association between NF-*κ*B and Inflammatory Cytokines as well as TGF-*β*1 and Histomorphometric Changes of Colon

**Figure 5:**
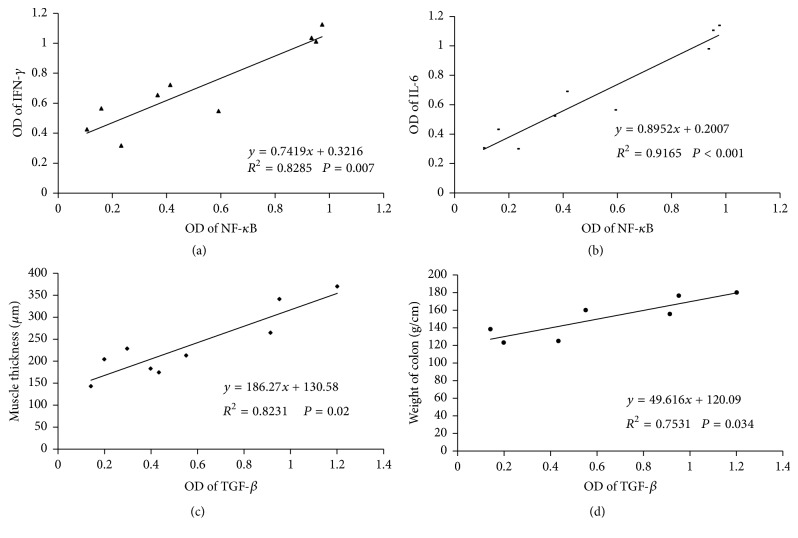
Correlation between NF-kB and IFN-*γ* (a), NF- kB and IL-6 (b), TGF-*β* and muscle thickness of colon, TGF-*β*, and weight per unit length of colon.

Linear regression analysis showed that the level of NF-*κ*B expression was associated with all inflammatory cytokines that were measured, including IFN-*γ* (Figure 5(a)), IL-6 (Figure 5(b)), TGF-*β*1, and Smad2/3 (Table 1). Moreover, level of TGF-*β*1 expression was associated with most of the histomorphometric parameters that were assessed, including mucosal and muscle thicknesses (Figure 5(c)), colonic wet weight (Figure 5(d)), and levels of collagens I and III in the mucosa. There was no significant association between the levels of collagens I and III in muscle layer with the level of TGF-*β*1 expression (Table 2).

## 4. Discussion

Morphology forms the basis of the function of human organs. GI thickening and stiffening occurs in diabetes [10–12]. However, the pathogenesis of GI thickening and stiffening is not well understood. Colonic motility is generated by the smooth muscle; therefore remodeling of its structure affects its contractile function [17]. The present study found that the muscle thickness of the colon increased in the DM group but this increase was inhibited in the TSF group. We also found that the number of smooth muscle cells per unit area in both circular and longitudinal smooth muscle layers was not significantly changed among three groups, which suggested that the increased smooth muscle thickness in smooth muscle layer of colon in diabetic rats should be due to the increased number of smooth muscle cells (hyperplasia) rather than due to the increased cell size (hypertrophy). We did not find noticeable microscopic damage in the smooth muscle cells and also did not find content changes in collagens I and III in the muscle layer in the DM group. There are evidences that the disorders of colon motility occurred in the diabetic patients [5, 18]. Our results may suggest that such motility changes may be, at least in part, due to remodeling of the smooth muscle layer and that damage of colonic motility is likely a contributing factor for constipation and diarrhea in diabetic patients [18]. In fact, we observed that the contractile ability of the colon is increased, and rhythm of contraction is disordered in type 2 diabetic rats (unpublished data). The present study showed that muscle thickness and contents of collagens I and III in the mucosal layer increased. It is well known that collagen accumulation is a main reason for sclerosis of tissues and organs; therefore accumulation of collagens I and III in the mucosal layer likely contributes to the stiffness of the colon wall in diabetic rats [1]. In our study, Tangshen Formula could inhibit collagens I and III from accumulating in the mucosal layer in type 2 diabetic rats, which suggest that this herbal compound is capable of attenuating complications of diabetic enteropathy. Increased expression of collagen is a result of infiltration of macrophages or activation of fibroblasts. The detail data in relation to the infiltration of macrophages or activation of fibroblasts and effect of Tangshen Formula on them will be observed in future study.

NF-kB is found in nearly all tissues and cells and its signaling pathway is important in the expression of proinflammatory cytokines, including IL-1, IL-6, IFN-*γ*, and TNF [19]. Since NF-*κ*B is involved in initiation of inflammation, its suppression could be effective for the treatment of inflammatory diseases [20]. NF-*κ*b has been shown to be closely related to the pathogenesis of diabetes. NF-*κ*b is the main target of excess glucose and oxidative stress; it then regulates the expression of genes related to inflammation [21]. In fact, diabetes is a state of chronic inflammation, which is associated with the occurrence of complications. Therefore, the thickening of gut wall in the diabetes may be closely related to the inflammation. Our present study found that expressions of NF-*κ*b, IL-6, and IFN-*γ* in the colon were dramatically increased in the DM group but were partly inhibited in the TSF group. Our previous study also indicated that TSF could inhibit expressions of NF-*κ*B, TNF-*α*, IL-1, and monocyte chemoattractant protein-1 (MCP-1) in the kidney of diabetic rats. Results of the present study suggest that TSF attenuates inflammation in the colon of diabetic rats, which might be attributed the thickening of gut wall in the diabetes.

TGF-*β* is a multifunctional polypeptide growth factor that inhibits GI inflammation by suppressing epithelial NF-*κ*B activation, maintaining levels of inhibitory *κ*B proteins (I*κ*B*α*) in the epithelium, and decreasing systemic serum levels of IL-6 and IFN-*γ* [22]. TGF-*β* also promotes intestinal epithelial restitution, improves intestinal mucosa integrity, and increases extracellular matrix and integrin production in intestinal injury [23–25]. Furthermore, through activating the ERK 1/2 signaling pathway, TGF-*β* may enhance intercellular tight junctions (TJs) [26, 27], thus decreasing epithelial permeability and strengthening the epithelial barrier [23]. In diabetes, the intestinal tract is in a chronic inflammatory state and the TJs are weakened, thus increasing epithelial permeability. Our present study showed that expression of TGF-*β*1 markedly increased in the colon of type 2 diabetic rats. The increased expression of TGF-*β*1 should be, at least in part, a negative feedback reaction to the dramatic increase in NF-*κ*B and proinflammatory cytokines. Our results demonstrated that the level of NF-*κ*B was closely associated with the level of TGF-*β*1. Thus, increased expression of TGF-*β*1 in diabetic rats appears to attenuate diabetes-induced increases in intestinal inflammation and epithelial permeability. However, overexpression of TGF-*β*1 can stimulate production of extracellular matrix and induce fibrosis in various tissues [28]. Our results indicated that expressions of TGF-*β*1 and Smad2/3 significantly increased with concomitant increases in collagens I and III in the intestinal tract of the DM group. We also found that the level of TGF-*β*1 expression was associated with levels of collagens I and III in the mucosa. These results suggest that overexpression of TGF-*β*1 is responsible for thickening and stiffening in the colon of diabetic rats.

Of interest, our results indicated that overexpressions of TGF-*β*1 and Smad2/3 could be inhibited by administration of Tangshen Formula (TSF). However, the mechanism by which TSF suppresses the increase in TGF-*β*1and Smad2/3 in the diabetic rats is not clear. Ohga and others reported that, under diabetic conditions, activated NF-*κ*B translocates into the cell nucleus and triggers expression of its target gene, besides the proinflammation cytokine, TGF-*β*1 [29], a negative feedback regulation likely responsible for overexpression of TGF-*β*1. Emodin is an active constituent in rhubarb, one of the herbs in TSF. Emodin has been found to stabilize I*κ*B*α* to arrest p65 nuclear translocation and inhibit the DNA-binding and transcriptional activities of NF-*κ*B, thus suppressing high glucose-induced TGF-*β*1 overexpression in mesangial cells [30]. Another herb in TSF is astragalus, whose active constituent is astragaloside IV, which has been shown to inhibit NF-*κ*B overexpression in the kidney of streptozotocin-induced diabetic rats [31]. The effects of these 2 constituents may be a mechanism by which TSF suppresses overexpressions of NF-*κ*B and TGF-*β*1, thus attenuating thickening and stiffening in the colon of diabetic rats. However, TSF is composed of 7 herbs, each with active constituents that exert biologic effects. Thus, the mechanism by which TSF affects the colon is more complex than the aforementioned individual effects of emodin and astragaloside IV. Exploration is needed to ascertain the precise molecular mechanism by which Tangshen Formula inhibits the increase in NF-*κ*B, TGF-*β*1, and Smad2/3 in diabetic rats. It also is interesting to know how TSF could inhibit the production of collagens I and III in the mucosal layer. The mechanisms by which TSF inhibits the production and deposition of collagen will be addressed in future studies.

In conclusion, increase in collagens I and III as well as thickening appears to be associated with upregulated expression of TGF-*β*1/Smad2/3 and may be responsible for biomechanical remodeling in the colon of type 2 diabetic rats. Moreover, the Chinese herbal medicine Tangshen Formula appears to attenuate colonic structure remodeling in type 2 diabetic rats possibly by inhibiting the overactivated pathway of NF-*κ*B and therefore reducing expression of TGF-*β*1/Smad2/3.

## Figures and Tables

**Table 1:** Correlation between NF-kb and inflammatory cytokines.

Parameters	Linear equations	*R* ^2^	*P*
TGF-*β*1	*y* = 0.8895*x* + 0.0981	0.7054	0.030
Smad2	*y* = 1.8986*x* − 0.1802	0.7355	0.001
Smad3	*y* = 2.0776*x* − 0.2421	0.8076	<0.001
Il-6	*y* = 0.8952*x* + 0.2007	0.9165	<0.001
IFN-*γ*	*y* = 0.7419*x* + 0.3216	0.8285	0.007

**Table 2:** Correlation between TGF-*β*1 and histomorphometric changes of colon.

Parameters	Linear equations	*R*^2^	*P*
Colonic weight (g/cm)	*y* = 49.616*x* + 120.09	0.7531	0.034
Mucosa thickness (*μ*m)	*y* = 109.76*x* + 184.15	0.6405	0.045
Muscle thickness (*μ*m)	*y* = 186.27*x* + 130.58	0.8231	0.020
Collagen I in mucosa	*y* = 0.0125*x* + 0.0164	0.6658	0.037
Collagen I in muscle	*y* = 0.0102*x* + 0.0254	0.2879	0.132
Collagen III in mucosa	*y* = 0.0148*x* + 0.0183	0.6069	0.048
Collagen III in muscle	*y* = 0.0111*x* + 0.0278	0.3048	0.071
